# Health professional perspectives on systems failures in transitional care for patients with dementia and their carers: a qualitative descriptive study

**DOI:** 10.1186/s12913-015-1227-z

**Published:** 2015-12-18

**Authors:** Ashley Kable, Lynnette Chenoweth, Dimity Pond, Carolyn Hullick

**Affiliations:** School of Nursing and Midwifery, Faculty of Health and Medicine, University of Newcastle, University Drive, Callaghan, NSW 2308 Australia; Faculty of Health, University of Technology, 15 Broadway, Ultimo, NSW 2007 Australia; Centre for Healthy Brain Ageing, University of New South Wales, G27, Botany Rd, Randwick, NSW 2031 Australia; School of Medicine and Public Health, Faculty of Health and Medicine, University of Newcastle, University Drive, Callaghan, NSW 2308 Australia; Hunter New England Local Health District, Rankin Park, NSW 2287 Australia

**Keywords:** Dementia, Discharge planning, Health care professionals, Research, Qualitative, Focus groups, Qualitative analysis

## Abstract

**Background:**

Healthcare professionals engage in discharge planning of people with dementia during hospitalisation, however plans for transitioning the person into community services can be patchy and ineffective. The aim of this study was to report acute, community and residential care health professionals’ (HP) perspectives on the discharge process and transitional care arrangements for people with dementia and their carers.

**Methods:**

A qualitative descriptive study design and purposive sampling was used to recruit HPs from four groups: Nurses and allied health practitioners involved in discharge planning in the acute setting, junior medical officers in acute care, general practitioners (GPs) and Residential Aged Care Facility (RACF) staff in a regional area in NSW, Australia. Focus group discussions were conducted using a semi-structured schedule. Content analysis was used to understand the discharge process and transitional care arrangements for people with dementia (PWD) and their carers.

**Results:**

There were 33 participants in four focus groups, who described discharge planning and transitional care as a complex process with multiple contributors and components.

Two main themes with belonging sub-themes derived from the analysis were:

*Barriers to effective discharge planning for PWD and their carers - the acute care perspective:* managing PWD in the acute care setting, demand for post discharge services exceeds availability of services, pressure to discharge patients and incomplete discharge documentation.

*Transitional care process failures and associated outcomes for PWD – the community HP perspective:* failures in delivery of services to PWD; inadequate discharge notification and negative patient outcomes; discharge-related adverse events, readmission and carer stress; and issues with medication discharge orders and outcomes for PWD.

**Conclusions:**

Although acute care HPs do engage in required discharge planning for people with dementia, participants identified critical issues: pressure on acute care health professionals to discharge PWD early, the requirement for JMOs to complete discharge summaries, the demand for post discharge services for PWD exceeding supply, the need to modify post discharge medication prescriptions for PWD, the need for improved coordination with RACF, and the need for routine provision of medication dose decision aids and home medicine reviews post discharge for PWD and their carers.

## Background

Previous studies have identified issues with the continuity of care following acute care discharge of people with dementia (PWD), in particular the lack of follow-up care services and the poor clinical outcomes that result [1]. Carers of PWD are critical to achieving successful transitional care and depend on clearly devised and documented discharge plans. Family support of PWD can, therefore, be hampered by inadequate service planning and continuity between acute, community and residential care settings. Post discharge, the carer role extends beyond providing care in the home; carers also administer medications, arrange health appointments, provide transport to health service providers and coordinate access to and timing of services in the PWD’s home and in community services [2]. In addition, carers most often take responsibility for recognising and responding to triggers that exacerbate confusion and agitation for PWD; and for communicating information to health professionals to assist them in assessing and communicating with the person (including information on medical history, usual behaviour, stressors, management strategies, and planned services).

Although there may be a range of services planned for PWD, none of them is available around the clock. If the person is discharged back to their home in the community, their carer is expected to take responsibility for their physical, emotional and social care and medical follow-up, often under trying circumstances [3]. Carers may be dealing with a person who wanders away from home, is active during the night and is significantly cognitively and functionally impaired. The effect of this situation can be carer stress, sleep deprivation, exhaustion, exacerbation of health problems, depression and anxiety [4].

The issue of how best to provide health and care services for PWD has come to the fore in Australia, with more than 342,000 people living with dementia in a population of 23 million [5]. The hospitalisation of PWD is particularly at issue, since 30 % of people over 65 years of age who represent 48 % of patient days in hospital 2011–2012, [6] have a cognitive impairment [7]. The number of people hospitalised having dementia as their principal diagnosis increased by 19 % between 2004–05 and 2009–10 [8]. Few dementia-specific discharge programs have been established to ensure their safe transition from hospital to home [2].

The Transition Care Program (TCP) designed to help older people in hospital return safely to their homes has been evaluated as having limited suitability for PWD, since it requires that the person has the capacity to make decisions, set goals and comply with prescribed therapy and health regimens [8]. When the person and/or their carer is expected to assume an active role in the transmission of information between different health service providers, or to follow and coordinate a treatment or care plan, there is a high risk for them to have an unsatisfactory transition between acute and community care, a post discharge adverse event and subsequent readmission to acute care [9, 10]. An adverse event is ‘an unintended injury or complication which results in disability, death or prolongation of hospital stay, and is caused by health care management rather than the patient’s disease’ [11]. Health professionals in acute care and primary care can undertake post discharge planning and follow-up strategies designed to minimise risks and associated poor outcomes during transitional care, and studying this process can inform the adoption of suitable strategies by health professionals.

A review of 73 studies on communication and information transfer between hospital and primary care physicians identified some major deficits in these processes. Direct communication between these physicians was infrequent (3-20 %) and availability of a discharge summary at the first post discharge visits was 12-34 %. These factors affected the quality of care in approximately 25 % of follow-up visits [12]. In addition, the quality of documentation on discharge summaries was poor, resulting in missed test results (33-63 %), inadequate treatment or hospital course details (7-22 %), non-provision of discharge medications (2-40 %), no test results pending at discharge (65 %), no patient or family counselling (90-92 %) and no follow up plans (2-43 %) [12]. Inadequate discharge documentation can give rise to misinformation, duplication of tests or interventions, delayed or failed referrals and potentially, patient harm [9, 13].

A systematic review of 18 studies that measured the association between continuity of care and patient outcomes, found that increased provider care continuity is associated with improved patient outcomes and satisfaction [14]. A pressing issue associated with poor hospital discharge for the person with dementia and/or their carer is the failure to ensure that there is a clear understanding of their medication regimen. These people are often discharged with their current medicines and instructions for use, but inadequate time is taken to ensure that the person and/or their carer understand the revised medication regimen. The person and carer are often unsure about how the pre-hospital medication regimen aligns with the current medication regimen, or whether the current or previous regimen needs to be followed [15]. In addition, people with dementia like many other Australians may also use non- prescription agents, herbal preparations and other over-the-counter preparations that may be contraindicated, or reduce the therapeutic action of their prescription medicine [16].

A recent study of medication reconciliation for people over 64 years of age discharged from hospital to the community reported that 70 % did not understand the new dosing instructions at discharge [15]. Given this finding, assessment by community health staff or pharmacist home medicines review after discharge can help to determine the person’s and the carer’s understanding of medication requirements and regimens, and also help to identify issues with use of non-prescription medicines.

These and other failings in hospital discharge for PWD are an issue of concern for their carer and health staff who provide services for them in community and residential aged care facility settings (RACF) [1]. Effective transitional care for the older person between different health sectors is, therefore, one way of ensuring their post discharge health, function and well-being, especially if they have a cognitive impairment [1]. Transitional care programs are designed to ensure the coordination and continuity of health care as the vulnerable person transfers between different levels and types of care [17]. Discharge planning should include a comprehensive package of coordinated care and detailed communication in the discharge summary about the reason for admission to acute care; treatment and assessments undertaken; arrangements made for follow-up services after discharge; and ongoing medication and management requirements [1].

Effective discharge planning for PWD, therefore, needs to involve community-based health professionals, including general practitioners (GPs), GP practice nurses, community nurses and other community service personnel, RACF staff where relevant, and the carer/s of the PWD. Acute care health professionals involved in discharge planning ideally commence this process from the time the person is admitted to hospital. Frequently the primary focus of this planning is in response to the person’s admission diagnosis and the need for continuity of care following discharge. For PWD it is equally important for post discharge planning to include management of symptoms, as well as ongoing issues faced by their carer/s, and their future health care needs in the context of dementia. The health and well-being of PWD and their carers following hospital discharge is sensitive to the transitional care processes established between the various health sectors [1] that involve health professionals in hospital and community settings. This study aimed to describe health professionals’ perspectives on discharge planning and transitional care for PWD and their carers in a regional area of Australia.

## Methods

### Design

A descriptive, exploratory, qualitative research design was used to conduct focus group discussions with health care professionals involved in and impacted by, discharge care planning and the provision of transitional care services for people with dementia (PWD). [18, 19].

### Ethics

Ethical approval was granted by the Hunter New England Health Human Research Ethics Committee and the University of Newcastle Human Research Ethics Committee (Approval numbers: 13/08/21/5.10, H2013-0333).

### Participants and recruitment

Purposive sampling was used to recruit volunteer health staff who were involved in discharge planning and transitional care activities in the acute care setting (including five junior medical officers (JMO) and 16 nurses and allied health professionals who were involved in discharge planning in one acute tertiary facility); and the community care setting (including eight GPs and GP practice nurses, and four staff in the residential aged care setting) involved in delivering transitional care. Potential participants were approached either by email or face to face by clinicians from four professional groups (who agreed to contact colleagues for this purpose) and invited to participate, and an information statement about the study and consent form were provided. We were unsuccessful in recruiting geriatricians, other physicians and subspecialist doctors to participate in a focus group.

### Data collection

A semi-structured focus group schedule developed by the research team framed the focus group discussions. Questions were derived from the literature on the discharge process and discharge of PWD and were checked for suitability by clinicians involved in discharge procedures. A study advisory committee was established which comprised representatives of the four professional groups and dementia carers. This committee provided comments and advice about the focus group questions, including appropriate wording and topics and suitable focus group structure (acute care and community care groups).

Focus group discussions were conducted at workplace meeting rooms at times that were convenient to the participants during the period December 2013 - April 2014, with sessions lasting between 35–90 min. Experienced members of the research team facilitated the focus group discussions (AK, DP) using the same questions to ensure consistency in questioning style. Discussions were digitally recorded, transcribed verbatim and de-identified in the written transcripts.

### Data analysis

Data were analysed independently by two members of the research team (AK, LC). Data were grouped into major content categories and further analyses were conducted using a structured approach described by Sandelowski [19] and Neergaard et al. [18], following the approach described by Miles and Huberman [20], as suitable for analysis of qualitative descriptive data (see Table 1).

**Table 1:** Data analysis

Analytic Strategy	Findings/Themes
Coding and recording reflections on data transcripts	2 Perspectives:
	1 Acute care perspective (pre discharge, ONE public tertiary facility).
	2 Primary care/RACF perspective (post discharge, from multiple acute care facilities).
Sorting data to identify topics	Topics: Discharge planning, cognitive impairment, carer involvement, communication and information, assessment, safety, discharge summaries, documentation, medications, post discharge services needed, key people for communication, multidisciplinary, carer needs, GP needs, RACF needs, PWD exacerbations in acute care, pressure on staff, time, expectations, Webster packs, discontinuity of care, access to services, insufficient information, outcomes
Identify categories and themes	Four main themes identified:
	1 Discharging PWD – The Process (not reported in this paper)
	2 Barriers to effective discharge planning for PWD and their carers
	3 Transitional Care process failures and associated outcomes for PWD
	4 Factors that would facilitate effective transitional care for PWD (not reported in this paper)
Identifying commonalities and differences among data	Commonalities: Complexity, variation in processes, multiple key stakeholders, patient safety, tensions between health staff in acute and community settings.
	Differences: Acute care HP and Community Care HP Perspectives about transitional care for PWD and their carers.
Deciding groups and generalisations that are true for the data	1 Contrasting Pre and post discharge perspectives (Acute care vs Community health professionals)
	2 Processes and stakeholders needs are variable and complex
	3 Barriers to continuity of care occur in acute and community settings
	4 Transitional care process frequently fails and results in poor outcomes for PWD and their carers
Examining generalisations in the light of existing knowledge	Consideration of results in comparison with previous studies.

The results of independent data categorisation were discussed and compared by two researchers (AK, LC) to determine the main themes reported by participant groups and the relationships between them were agreed.

## Results

Participant numbers and characteristics are reported in Table 2. The size and number of groups was due to the availability of staff to participate at a time and place convenient to them. Each group was convened during scheduled breaks in their working day, and the participants displayed a high level of interest in the topic and actively engaged in the focus group discussions. Acute care participants were from one tertiary acute facility, and general practice and RACF participants received discharged PWD from several public and private facilities.

**Table 2:** Participant numbers

Focus groups	Number of participants
Acute Care	
1 Junior medical officers	5
2 Discharge planners including: nurses, allied health staff, clinical nurse consultant in community liaison and dementia	16
Community Care	
3 Residential aged care staff	4
4 General practitioners, practice nurses and practice administrators	8
Total participants	33

Two major themes were identified from the analysis of data: Barriers to effective discharge planning for PWD and their carers from the acute care perspective; and Transitional care process failures and associated outcomes for PWD from the community perspective. Subthemes were derived from these two themes.

### Barriers to effective discharge planning for PWD and their carers: the acute care perspective

#### Managing PWD in the acute care setting

Acute care staff frequently encountered episodes of confusion and agitation in the PWD struggling with the unfamiliar hospital environment. They reported that carers described these episodes as being ‘different from their normal behaviour’, and were considered to be consistent with an exacerbation of symptoms.

‘…. the carer will say, ‘no, this is not what they are normally like at home’ (DP group).

Staff described having difficulty managing PWD because of the lack of a specialised unit to manage PWD in the acute care setting, and did not appear to recognise that the confusion seen in PWD in hospital was most likely their response to the unfamiliar and over-stimulating environment.

Every ward you go to in the middle of the night has one or two dementia patients and nurses are . . . calling us telling us to chemically sedate them or hold them down because . . . they don’t know how to manage . . . if they had expert people that specialise in that area and just looked after these (patients), things would be a lot smoother (JMO group).

#### Demand for post discharge services exceeds availability of services

Discharge planning staff encountered significant obstacles when planning transition to community services for PWD, including accessing services and negotiating service criteria. They reported it was difficult to find a service that could deliver all the care required.

The barriers are getting services! That’s the major challenge . . . Getting services is becoming really hard so unless they’re eligible for ComPacks, (short term non clinical case managed packages of community care [21]), . . . the supply is a lot less . . . than demand. There’s lots of restrictions (DP group).

Participants also described problems with accessing post discharge services for PWD because of long waiting lists for these services and were concerned about whether the referral remained active, and whether the referred service would be delivered.

. . . a long waiting list for access to community physio, . . . day hospital, could be weeks. . . we’ve had people . . . waiting for up to 2 years . . . if the service hasn’t come after 30 days they drop off that list and they’re not in the system (DP group).

In addition, participants described clinically significant concerns about the cognitive and functional safety of PWD after they were discharged home. They were concerned that community-based services were often delivered intermittently and that the carer was left to assist PWD at other times, if indeed there was a carer living with them.

Even if you can get services (for) 3 h a week what happens . . . for the other 23 h of the day when there’s no one coming in? . . . Cognitive problems (are) . . . 24 h problems and there’s very few 24 h services (DP group).

Participants advised that, at times, the rehabilitation service provider would not provide a service to PWD if their cognition is poor as a diagnosis of dementia excludes them from being eligible for the service. Some acute care staff advised that they deliberately do not test cognition in PWD prior to discharge, to increase their chances of gaining access to rehabilitation services. Discharge planning staff also observed that although services may have been arranged for PWD, the PWD may refuse them at the time the service provider attempts to deliver them.

#### Pressure to discharge patients and incomplete discharge documentation

Acute hospital staff described experiencing significant pressure to discharge PWD early and at short notice. This pressure caused added stress to the nurses and allied health staff involved in discharge planning, particularly when they considered that the person was not safe to be discharged.

The onus is to get patients out of hospital . . . once you identify that they’re safe for discharge it’s . . . get them out as quick as possible, there’s a lot of pressure (JMO group); . . . there is that push to get them out the door really quickly . . . last year our expectation was to discharge 5 or 6 patients a day. It’s now up to 7, 8. Last week Friday we discharged 13 patients . . . so it’s push, push, push . . . (DP group).

Participants explained that there are up to three discharge summaries documented for PWD: the medical discharge summary, the nursing discharge summary and the allied health discharge summary. The primary purpose of the medical discharge summary is to communicate relevant information to the GP to assist them to provide appropriate post discharge treatment and services. This document is also provided to the patient or their carer, which may not be appropriate for PWD, because information about their cognitive impairment may cause distress.

Staff involved in discharge planning advised that the nursing and allied health discharge summaries frequently contain valuable information about how the patient and their carer copes at home and includes information about the services they need to support them at home. However, these documents are not consistently provided to the GP or the RACF staff. Also, in some instances JMOs or allied health staff are required to attend to more acutely ill patients as a priority, and consequently PWD may be discharged before they can be seen by these health professionals.

. . . if . . . you could sit down (with each patient) and say this is why you were here, this is the plan when you leave, but it actually happens very rarely (JMO group).

JMOs also stated they did not have guidelines for discharge planning of PWD. Some JMOs stated that when they write the medical discharge summary, they often find they do not have sufficient information about PWD, from the medical records, or from their senior colleagues, in order to plan care for PWD. This can result in a discharge summary that addresses ongoing treatment on medical issues, but without consideration of the person’s dementia-related needs.

. . . people doing the discharge summaries are the ones that have the least comprehensive overview of what’s going on, . . . don’t really have a clear sense of what the patient came in with, and the main issues . . . so you do it from the notes but notes aren’t always amazing . . . you just hope you have a good registrar and a good consultant and some very supportive nurses that can help you (JMO group).

The documentation and reconciliation of medications in the discharge summary was described as quite challenging for the JMOs, because they could not always determine which medicines were permanently ceased, which medicines should be changed at the time of discharge and which were new, or should be recommenced. The JMOs advised that medicines not directly relevant to the patient’s reason for admission can easily be overlooked. In addition, the hospital pharmacy did not have dose administration aids such as Webster packs for their medicines. This was considered to be a significant problem for PWD because of difficulties with memory and orientation (day and time) and when the person is taking many medicines (polypharmacy) at different times.

### Transitional care process failures and associated outcomes for PWD: the community hp perspective

The barriers to effective transitional care described above were reported to result in process failures and poor outcomes for PWD.

#### Failures in delivery of services to PWD

Failures in care for PWD in the acute care setting and after discharge were reported by health professionals who were following up PWD after discharge from acute care. RACF staff described carers’ reports that confusion was frequently managed with chemical and physical restraints during hospitalisation.

They physically restrained her . . . she actually chewed through one of the restraints, lost a couple of teeth, climbed over the bed rail and fractured her other hip (RACF group).

Where acute care staff were unable to reduce the confusion or agitation occurring in PWD during hospitalisation, PWD were considered to be at risk of poor outcomes such as having falls and/or being overmedicated. In addition, RACF staff described PWD being discharged without their health issue on admission being treated and considered that in these cases PWD were being treated disrespectfully by acute care staff.

. . . send them for chest pain, they get diagnosed with a urinary tract infection and dehydration . . . they’re treated like second class citizens (RACF group).

Inadequate documentation of discharge plans from the acute care setting was reported to result in non-delivery of some planned services in the community. Other reasons cited for the failure of follow up community services for PWD included long waiting lists and access and transport problems. For example if ambulance transport was required, the PWD may wait for hours for a service due to the transport schedule being different to the timing of the appointment.

#### Inadequate discharge notification and negative patient outcomes

RACF staff consistently reported not being advised of a patients imminent discharge, or only receiving short notice, not receiving adequate discharge documentation, not receiving Webster packs of prescribed medicines (resulting in missed or delayed administration), being pressured to accept discharges after hours and often not receiving any information about new equipment needed for a patient prior to discharge (eg bariatric equipment). These deficits meant that RACF staff were inadequately prepared to receive and care for PWD following discharge, especially in relation to workforce capacity.

We had someone sent back that week and they didn’t bother ringing, they just got the ambulance transport and got them here at two o’clock in the morning (RACF group).

On some occasions, when RACF staff were notified about a patient due to be discharged, they described experiencing significant pressure from acute care staff to accept the person. This frequently occurred at short notice, often late in the day or at night when qualified staff were not on duty to supervise safe care for the person. RACF staff noted that this practice is contrary to the policy “Care Coordination: From Admission to Transfer of Care in NSW Public Hospitals”, that states low-level care residential facilities are unable to accommodate patient transfers after hours because they do not have qualified staff to manage the care involved, and that it is not appropriate to transfer patients to these RACFs after 4 pm or overnight [22].

. . . the NUM (Nurse Unit Manager) . . . said “What? You’re refusing?”, And I said, “Yes I am” and she said, “and what right have you got?” and I said, “because we’re a low care facility, we haven’t got an RN here (RACF group).

These issues associated with discharge notification further reflect a lack of understanding of the pressure and challenges for health care professionals on both sides of the discharge process. RACF staff who did not receive adequate information or have adequate staff after hours, recognised that PWD were at risk for missed treatment and medications and poor outcomes.

#### Discharge-related adverse events, readmission and carer stress

PWD and their carers were often not provided with essential information about their planned transitional care. The communication provided to the carer (about follow up arrangements and contingency plans) was often inadequate, because the patient was discharged before being seen by some members of the acute care team.

GPs had concerns about the inexperience of the JMOs writing medical discharge summaries, given the vital information that was often missing from discharge summaries.

. . . should the discharge summary be written by the most inexperienced person? I think it’s completely inappropriate . . . overall we need less biomedical information and more . . . psychosocial information, . . . discharge letters usually don’t indicate that they have showers 3 times a week and somebody comes and cleans and checks on their Webster pack (GP group).

The GP may not know the patient is being discharged, and the patient may be presenting to them for the first time, so they need the entire medical history because it can take four weeks to access primary care notes from another practice. GPs described receiving discharge documentation that contained a lot of irrelevant information, did not always include ACAT (Aged Care Assessment Team) assessments, or inpatient assessments of cognition that may be irrelevant when the patient returns to a familiar environment.

If there’s been an ACAT assessment done (and) . . . sent to the GP so that they know exactly what that person is entitled to, what they’ve been approved for (services); . . . what happens is you get an ACAT assessment of the person who was in hospital with dementia out of their . . . comfort zone, in an unfamiliar routine and the person who presents in your nursing home never looks like the person who is on that piece of paper. You would swear you’ve got two different people (GP group).

Subsequent management of PWD by GPs can place them at risk for adverse events if post discharge services are poorly aligned with their needs; and this can increase carer stress.

Similarly, the discharge documentation provided to RACF staff was frequently inadequate (usually only a nursing discharge summary) and information about the patient’s medicines and associated orders were frequently inadequately documented.

It is extremely rare for an aged care facility to even get a discharge summary (GP group) . . . we call the hospital and quite often they haven’t written it yet . . . say they will send it to the doctor and then the doctor might have to chase it up a few days or a week later. . . We didn’t know she was coming back . . . there was a nursing discharge summary, no medical discharge summary and photocopies of . . . the medication chart (RACF group).

Discharge documentation deficits contribute to discontinuity of care, adverse events, readmission and associated carer stress.

#### Issues with medication discharge orders and outcomes for PWD

GPs indicated that they routinely revised planned discharge treatment orders, particularly medications. They noted excessive prescribing, to manage confusion and agitation in acute care that resolved when the person with dementia returned to their normal environment, and that these medications were no longer required.

. . . poly-pharmacy more than five . . . is a disaster . . . my first step invariably is to cross out half of the stuff that doesn’t make any sense . . . and then see the patient (and) cut out the other half. . . and usually the patient gets better. They’re just bombed out of their mind over and above the cognitive deficiency they truly have (GP group).

In addition, the GPs stated that they needed, but rarely received, information about medicines that had been ceased, changed, or commenced in the acute care setting, and reasons for these changes, as well as medicines to be continued.

. . . medication information . . . is critical . . . the rationale for changing medicines . . . you really need to know why . . . why they’ve suspended medication or why they’ve started a new one too but especially why they’ve stopped a medication (GP group).

Often incomplete copies of hospital medication charts were sent to the RACF which made it challenging for staff to ascertain which medicines were most recently administered and when.

So I can’t even tell what their last recent . . . medications were, what they’ve last been administered . . . She was previously on warfarin and they had ceased that. I couldn’t see why they had ceased it (RACF group).

In RACF (low care residential facilities), staff are only allowed to administer medications from dose administration aids, since many staff administering medicines do not have formal nursing qualifications. However, participants advised that when the resident returned to the facility, only boxed medications were provided. RACF staff frequently had to request the patient’s GP to provide prescriptions for the patient as soon as possible following discharge, and then arrange for the community pharmacist to fill these prescriptions in dose administration aids.

. . . it can lead to really significant poor outcomes for the resident . . . if they’re on antibiotics for an infection or some blood pressure medication . . . chances are they’re not actually going to get it . . . we’ve never once received a Webster pack (RACF group).

These process failures either individually, or in combination, constitute a limited understanding of discharge requirements and inadequate communication between health professionals working in different health settings, and with patients and their carers at the time of discharge. This can result in poor outcomes for vulnerable PWD, such as missed medicines, overdosing and possible readmission to hospital, as well as additional stress for carers, GPs and RACF staff.

## Discussion

Transitional care is focused on achieving coordination and continuity of care as patients are transferred between different care providers, to avoid readmission to hospital and optimise health, particularly for vulnerable older people [23]. When transitional care fragmentation and failures occur, vulnerable people such as those with continuous complex care needs and cognitive impairment are at risk for “falling through the cracks” [1, 24, 25]. There is no theoretical basis or framework available for evaluating and implementing transitional care programs [23]. There are two transitional care models that have been demonstrated to be effective from trials: the transitional care model (TCM) by Naylor [26], and the care transitions intervention (CTI) by Coleman [27]; and some consensus standards for transitional care have been developed [28]. However, a systematic review of transitional care programs has determined that most trials of transitional care programs exclude people with cognitive impairment and dementia and high risk older adults [25].

Previous studies and reviews have identified some essential components for effective transitional care. A study by Wee and Wrijhoef identified four components: identifying patients at high risk for readmission, in-hospital assessment and substantial contact, care planning and post-discharge engagement and follow-up with patients and carers [23]. Nelson identified four components in the Coleman model: Coaching in hospital, home visiting within 72 h of discharge, medication review and follow up phone calls; and additional components in the Naylor model: nurses in the role of clinical leader or care manager, patient assessment to identify high risk patients, team care planning, home visits, telephone outreach and accompanying the patient to the first primary care follow up visit [29]. Medication management and review, particularly where polypharmacy is involved, is considered to be critically important during care transitions [24, 25, 28, 29]. This study identified several previously unreported issues that demonstrate the complexity and challenges associated with achieving these components of transitional care for PWD and their carers.

### Issues in acute care

In this study acute care staff described encountering substantial pressure and barriers in their efforts to provide safe discharge planning for PWD. They had significant administrative pressure placed on them to discharge patients early and hospital staff expressed concerns about the risks and safety of PWD after discharge because of their cognitive and functional deficits, and the availability and capacity of the person’s carer. In addition, discharge summaries were mainly compiled by JMOs who generally had limited knowledge of the PWD during their hospitalisation. Consequently, critical information and understanding about the needs of PWD and their carers during transitional care was often lacking, with negative impacts for carers, GPs and RACF staff.

### Barriers to effective post discharge planning

Discharge planning was really difficult due to challenges associated with patient risk assessment and the lack of available services for PWD, specifically the need for 24 h services and support. The perspective of acute care discharge planners was that community services are limited, difficult to access, have rigorous criteria for eligibility for a service and often exclude PWD. The systems operating are often not adequate for the needs of PWD and their carers, and there may be long waiting periods for community services that result in delayed or missed treatment. Even when the acute facility has discharged PWD with ideal discharge summaries and follow-up plans, the chances of these plans being executed as intended are unlikely because of the complexities associated with post discharge service access. In addition, and possibly as a consequence of limited services, it was evident that there were tensions occurring between health staff involved in transitional care for PWD in acute, community and residential care settings.

### Issues in the community

One major issue described in this study was that when cognitive assessments were conducted during hospitalisation, the possible deleterious effect of the unfamiliar hospital environment and care practices, and the potential effects of any new medicines prescribed for the PWD during hospitalisation could be contributing factors to the resultant assessment score. Following discharge, the PWD often had some improvement in their confusion when they returned to a familiar environment, and needed modifications in prescribed medicines (dose reductions or cessation of medications).

Also in the context of high pressure discharge processes, the practice of transferring unwell PWD from hospital to low-care RACFs which have very high resident-staff ratios and limited access to qualified nurses, particularly out of hours, the scene is set for increased patient risk of an adverse event. This situation can be further complicated when discharged PWD are a new admission to RACFs from hospitals, where RACF staff don’t know them, and receive limited discharge information, particularly baseline information about the person’s cognitive, psychosocial and physical capabilities and requirements. The subsequent outcome for the PWD could be extremely negative and very stressful for their carer(s) [12] with an increased risk of readmission to hospital.

### Issues associated with medication management for PWD

Although previous studies have identified medication management as an important component of transitional care, this study has identified issues that are critically important for PWD and their carers (who may also have limited capacity to assist the PWD). The existing processes for provision of prescribed medicines at discharge are unhelpful for PWD, their carers and RACF staff. Routine provision of pharmacy filled medication dose decision aids would contribute to reducing medication risks for these people following discharge. Information provided to patients and their carers should include sufficient information about medications so they know which medicines to take, how to take them and how to obtain them, and should be based on a process of medication reconciliation to adjust for changes that have occurred during hospitalisation [12, 30, 31]. Improved continuity of care in medication administration, alone, has the potential to improve outcomes for PWD [2, 12, 14, 30]. In the United Kingdom, a toolkit has been recently released for the purpose of encouraging routine referral of hospital patients to community pharmacists for post discharge assistance with medications [32]. This initiative could substantially improve patient safety during transitional care.

This study aimed to describe health professionals’ perspectives on discharge planning and transitional care for PWD and their carers in a regional area of Australia. Although some of the issues described here have been reported in some previous studies [2, 33–36], few studies have recognised the impact on PWD due to their increased vulnerability. Previously reported limitations of time, resources, bed availability, administrative pressures and inadequate information about patient needs in the acute care setting, have major impacts on the discharge planning process [12, 35, 37, 38] and these can potentially result in more negative outcomes for PWD. Resulting outcomes can include: inadequate notification of impending hospital discharge to GPs and the RACF; inadequate documentation in discharge summaries and follow-up plans to enable preparation for and care of PWD after discharge; failed receipt of discharge summaries and follow-up plans by GPs and RACF; delayed or failed delivery of essential community services; deficits in information about medications provided, discontinued, changed or commenced with reasons for these changes; and PWD being given excessive amounts of medication for confusion and/or agitation while hospitalised. In addition, the carer will often need to assume the responsibility for managing and coordinating post discharge services and treatment for the PWD. Improved acute care discharge processes should be consistent with the recently introduced Framework for Integrating Care for Older People with Complex Health Needs [39] that includes transitional care; and should assist carers to access community based services and resources that can contribute to improved outcomes for PWD [36, 40].

Strengths and Limitations: The strength of this study includes giving voice to a range of health professionals with different perspectives about the discharge planning and transitional care processes for PWD in the acute, community and residential care contexts. A study limitation was that the voices of specialist physicians were not heard because work pressures prevented them from participating in a focus group or an interview. As the focus group data were provided by a volunteer sample of health professionals in only one region of Australia, the findings will likely be biased and non-representative. In addition, the relatively small sample size and the qualitative nature of the focus group data, may limit the transferability of the findings to other contexts where systems and processes are different. These data, nevertheless, signal the type of health system barriers and failures that need to be remedied to benefit PWD and their carers during hospital discharge and care transitions.

## Conclusions

Participants described discharge planning and transitional care for PWD as a complex process with multiple contributors and components. Discharge planners recognised that PWD are vulnerable because of their reduced ability to comprehend and act on discharge requirements, their inability to manage their own care and treatment post discharge and not understanding and/or remembering the events planned to occur on prescribed dates. Consequently, PWD are frequently unable to manage their own medications, other treatments and appointments, and follow discharge instructions. Since not all PWD have a carer, this can have profound implications for discharge planning and provision of services in the community. For PWD who do have a carer, the carer’s knowledge about their social and health history, their usual abilities and responses to stressful situations, and the nature of their psychosocial needs, can be critical knowledge to share with acute care staff in the discharge planning process. Carers can, subsequently, become ‘de facto’ care managers and coordinators in the delivery of community and primary care services following discharge from acute care. It was apparent that health care professionals described engaging in discharge planning and transitional care processes with good intentions, despite having many challenges. They identified significant issues and barriers to this process that can result in poor outcomes for PWD and their carers, and have negative impacts for GPs and RACF staff. This aspect of acute care services requires the attention of policy makers, hospital administrators and hospital staff who care for PWD. The critical issues identified in clinical practice in this study include: pressure on acute care health professionals to discharge PWD early, the requirement for JMOs to complete discharge summaries, the demand for post discharge services for PWD exceeding supply, the need to modify post discharge medication prescriptions for PWD, the need for improved coordination with RACF, and the need for routine provision of medication dose decision aids and home medicine reviews post discharge for PWD and their carers. Future studies should develop needs based innovation strategies to address these identified deficits in transitional care for PWD.
